# Missouri

**Published:** 1896-08

**Authors:** 


*Missouri.* St. Louis is receiving her primary lessons in dealing with tuberculosis among her dairy cattle, and the newspapers are teeming with criticisms of all shades and colors. The reliability of tuberculin as a diagnostic agent is raised with as much force as if it was so new a medium as to need further tests. A little longer, and they will realize that this whole work has been done long ago and its value settled as being beyond question the keenest and most reliable method known.

The new ordinance of St. Louis places all dairies in the city under its provisions; that the sanitary officers of the Health Department shall inspect all stables, barns, buildings, sheds, lots, and pastures where cows are kept or fed, the products of which are sold in the city; the daily cleaning of all milk depots and dairies, all cow stables, the weekly cleaning of all lots, and no draining of these places to any stream or water-course; proper facilities for cooling milk; it also makes provision for drainage of all future cow stables, and permits to erect the same must be obtained by municipal ordinance. An application to erect such building must be filed, stating location, name of applicant, number of cows to be provided for, size and dimensions of stable, the same of lot or field, whether same is contiguous to a city sewer, or by what method manure and refuse are to be removed, as to city water connections, and method of washing out stable. The dimensions of stalls, height of ceiling, character of floors, drains, ventilation and location, as to doors in connection with building line, are provided for. Alterations and rebuilding of old stables are provided for in the new requirements for light, ventilation, and drainage. Stables not complying with these requirements may be declared nuisances by the Board of Health, and the owners cited before the courts to answer therefor. Suitable fines and punishment are provided for the several violations enumerated.

The appointment of two practical veterinary surgeons as inspectors is provided for; salary $175 per month, and they shall provide themselves with horse and carriage; said veterinarians to give bond of $5000 for faithful performance of duty; bondsmen to be in no way connected with milk or dairy business. They shall have ingress and egress to all properties involved in the milk business, to examine into the physical condition of all milch cows. Penalties for interference with said inspectors, concealment of diseased animals, etc., are provided for. Duplicate reports of diseased ones are to be filed. Their condemnation and destruction are provided for. Suitable fines are provided for officers of the city failing to perform their duty; provision for quarantining dairies wherever smallpox, diphtheria, scarlet or typhoid fever may exist, prohibiting sale of milk, and requiring fumigation, etc., of premises before quarantine can be raised.

St. Louis is much agitated over the inspection of the cattle furnishing the milk supply of the city, and all sorts of rumors are floating about. The herd at the city's poor farm, largely Holstein in breed, exhibited twenty-two out of twenty-eight with tuberculosis under the tuberculin test. State Veterinarian White and Dr. Ellis, City Inspector, are giving the matter almost their entire attention. The lack of a compensating clause to the owners for animals condemned and destroyed is creating much opposition. Deaths among children from tuberculosis and intestinal troubles have been very numerous throughout the city, and the milk supply is charged with a great deal of responsibility therefor. The State and City Boards of Health are working together in this movement and are very determined.

